# Lambl’s excrescences in a 9-year-old Japanese boy: differentiation from infective endocarditis

**DOI:** 10.20407/fmj.2025-049

**Published:** 2026-05-14

**Authors:** Hidetoshi Uchida, Kazuyoshi Saito, Masayuki Hirai, Meiko Hoshino, Kayoko Takada, Yumiko Asai, Arisa Kojima, Akira Yamada, Tetsushi Yoshikawa

**Affiliations:** 1 Department of Pediatrics, School of Medicine, Fujita Health University, Toyoake, Aichi, Japan; 2 Department of Pediatrics, Kariya Toyota General Hospital, Kariya, Aichi, Japan; 3 Department of Cardiology, School of Medicine, Fujita Health University, Toyoake, Aichi, Japan

**Keywords:** Lambl’s excrescences, Papillary fibroelastoma, Infective endocarditis, Modified Duke criteria, Transesophageal echocardiography

## Abstract

Lambl’s excrescences (LEs) are filiform extensions arising on the closing surface of cardiac valves, typically occurring at coaptation lines of left-sided valves. LEs are more commonly reported in adults and pediatric cases remain rare, with no previously documented cases in Japan. A 9-year-old Japanese boy initially presented with a fever and sore throat but developed right-sided neck pain several days later. Blood tests showed a white blood cell count of 33,900/mL and C-reactive protein concentration of 9.95 mg/dL. Contrast-enhanced computed tomography showed a right peritonsillar abscess, and cefotaxime and clindamycin were administered. The patient’s symptoms persisted for 4 days. Therefore, he was transferred to our hospital. Transthoracic echocardiography showed trivial aortic regurgitation and a 4.4×1.0-mm filamentous structure attached to the aortic valve. The patient improved with antibiotic treatment alone, which was continued because infective endocarditis was suspected. However, transesophageal echocardiography showed no shape-like vegetations, papillary fibroelastomas, or thrombi, and repeated blood cultures were all negative, which led to a diagnosis of LEs. After completing a 3-week antibiotic course, the patient was discharged and remained asymptomatic with no structural changes in the LE in 2 years. To the best of our knowledge, this is the first report of a pediatric case of LEs associated with aortic regurgitation in Japan, highlighting that LEs should be considered in children with aortic regurgitation.

## Introduction

Lambl’s excrescences (LEs) are filiform extensions arising on the closing surface of cardiac valves, and they are typically approximately 1 mm wide and occur at coaptation lines of the left-sided valves. LEs arise from fibrin deposition at sites of mechanical endocardial injury and from the proliferation of endothelial cells around them because of shear stress on the valve surface caused by turbulent blood flow, such as that associated with systolic ejection flow or regurgitation flow. In response, fibroblasts and fibrinogen are recruited for tissue repair. Over time, the repeated cycle of injury and healing leads to the gradual formation of a string-like fibrin mass, often referred to as a “wear and tear” process.^1,2^

In adults, the prevalence of LEs is approximately 5.5% in the general population as shown by transthoracic echocardiography (TTE).^1^ LEs can be observed in healthy individuals, and aortic regurgitation (AR) and mitral valve regurgitation (MR) do not always coexist. Additionally, cases of cerebral embolism associated with LEs have been reported.^3^ A definitive diagnosis requires differentiation from infective endocarditis (IE) (vegetation) and papillary fibroelastoma (PFE). The prevalence of LEs in children is reported to be 0.9%–2.6%, which is less than that in adults, and it is often discovered incidentally during TTE.^1^ The most common site of detection of LEs is the aortic valve. LEs are asymptomatic and follow a benign course even during long-term follow-up.

However, during the progression of LEs, fragments of the fibrin mass may detach or thrombi may form at the lesion site. If these embolic fragments enter the systemic circulation and occlude cerebral blood vessels, they can cause cerebral infarction.^4^ Internationally, the first pediatric case of LEs complicated by cerebral infarction was reported in 2022,^3^ but no pediatric cases of LEs have been reported in Japan to date. Here we report a case of a 9-year-old Japanese boy with Lambl’s excrescences that required differential diagnosis from infectious endocarditis,

## Case Presentation

A 9-year-old Japanese boy initially experienced a fever and sore throat, which resolved spontaneously. However, 3 days later, he developed a recurrent fever and right-sided neck pain. He visited a local clinic because his symptoms persisted and there was onset of torticollis and difficulty opening his mouth,. He was diagnosed with pharyngitis and prescribed antibiotics, but his symptoms did not improve. The next day, he was referred to a Pediatrics Department at a general hospital, where he was admitted for further examination and treatment. Blood tests showed a high level of inflammation, with a white blood cell count of 33,900/mL and C-reactive protein concentration of 9.95 mg/dL. A contrast-enhanced computed tomography (CT) scan detected an abscess in the right parapharyngeal space, which led to the diagnosis of a right peritonsillar abscess. Antibiotic therapy with cefotaxime and clindamycin was initiated.

By the fourth day of hospitalization, the boy’s clinical condition improved, with a decrease in the white blood cell count to 17,600/mL and C-reactive protein concentration to 6.91 mg/dL. However, a follow-up CT scan showed no improvement in the abscess, which raised concerns about descending mediastinitis. Therefore, he was transferred to our hospital for further management and possible abscess drainage.

The patient had no major medical or family history, including Marfan syndrome, and had not undergone dental procedures in the preceding months. However, a known allergy to penicillin was noted.

Physical findings at the time of admission (day 1) were as follows: height, 143 cm (+1.5 Standard Deviation); weight, 33.2 kg (+0.2 Standard Deviation); body temperature, 37.6°C; blood pressure, 111/74 mmHg; heart rate, 68/min; respiratory rate, 18/ min; and SpO_2_, 97% (room air). Neither an ejection click nor a marked systolic murmur except Lev 1/6 diastolic murmur at the second left sternal border was observed on auscultation in the thorax. No abnormalities were observed in the abdomen, and there was no skin rash and no Osler nodules on the palms and soles. Redness, swelling, and pain in the pharynx and neck pain were observed, although these symptoms were gradually improving.

**Figure 1:**
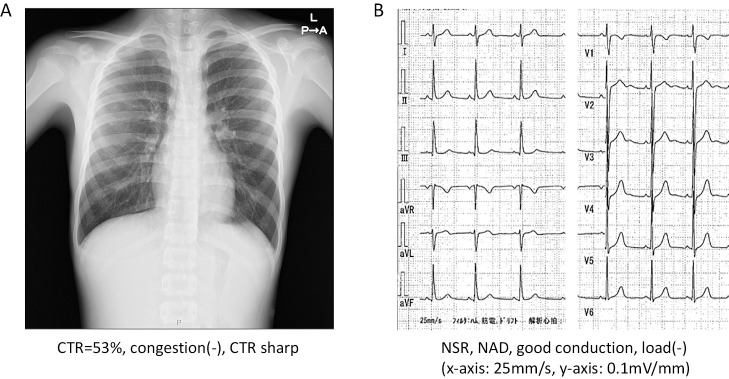
Chest X-ray and electrocardiographic findings. (A) Chest X-ray showing a cardiothoracic ratio (CTR) of 53%, with no signs of congestion. The costophrenic angles (CTR) are sharp, and bone and soft tissue structures appear normal. (B) Twelve-lead-lead electrocardiogram showing a normal sinus rhythm (NSR), a normal axis (NAD), good conduction, and no evidence of cardiac overload or ST-T wave abnormalities.

A chest X-ray showed no cardiac enlargement, congestion, or consolidation (Figure 1A). Electrocardiography revealed a normal sinus rhythm, a normal axis, good conduction, no signs of cardiac overload, and no T-wave abnormalities (Figure 1B). Blood tests showed a renewed increase in the inflammatory response, with a white blood cell count of 10,800/mL, neutrophil count of 74.8%, and C-reactive protein concentration of 3.65 mg/dL, though this was less pronounced than previously. A venous blood gas analysis and urinalysis results were normal (Table 1).

On the basis of the findings that the fever had resolved, clinical symptoms were improving, and there were no signs of active airway compression in the CT scan performed by the previous hospital, the antibiotics were determined as being effective, and medical treatment was continued.

TTE was performed and showed a thread-like structure of 4.4×1.0-mm, which was attached to the commissure between the right coronary cusp (RCC) and left coronary cusp (LCC) of the aortic valve. The morphology of the aortic cusps was normal with a balanced tricuspid valve. The dimensions of the aortic valve annulus, sinus of Valsalva, ST junction, and ascending aorta were 18.0 mm (+0.406z), 24.0 mm (+0.053z), 18.0 mm (–0.794z), and 19.4 mm (–0.027z), respectively. No prolapse or cleft of the aortic valve was observed. The dimension of the left ventricle (LV) was within the normal range (Left Ventricular end-Diastolic dimension=42 mm, Left Ventricular end-Systolic dimension=23 mm, and Left Ventricular Ejection Fraction=68%).

**Figure 2:**
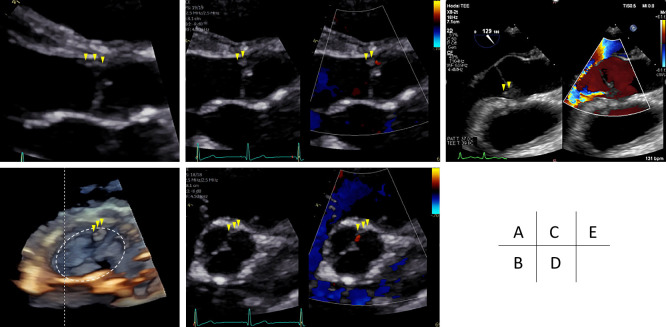
Transthoracic and transesophageal echocardiographic findings. (A) Long-axis (LAX) view of the aortic valve (AoV) on TTE showing a 4.4×1.0-mm thread-like structure attached to the line of valve closure (yellow arrow heads). The structure appears to go back and forth between the left ventricular (LV) side and the aortic side. (B) Three-dimensional echocardiographic view from the LV side showing a Lambl’s excrescence (LE) (TTE). The LE arises from the commissure between the right coronary cusp (RCC) and left coronary cusp (LCC), projecting toward the LV side after AoV closure (yellow arrow heads). (C) LAX view and (D) short-axis view of the AoV on transthoracic echocardiography (TTE), with color Doppler imaging showing mild aortic regurgitation (AR) originating near the commissure of the RCC and the LCC. A structure adheres to the same site (yellow arrow heads). (E) LAX of the AoV on transesophageal echocardiography (TEE) (mirror image of panel C).

The structure moved back and forth between the LV side and the aortic side. Trivial to mild (grade 1) AR was also observed at the same commissure site (Figure 2, S-movies 1–3).

In consideration of the possibility of IE, antibiotic therapy was continued. TEE was performed and showed an AR jet from the middle of the aortic valve to the RCC-LCC commissure. However, vegetation, a round abscess on the valves, or thrombus in the left atrial appendage was not observed (S-movie 4). A head magnetic resonance imaging scan was also performed, but no cerebral hemorrhage, cerebral infarction, or brain abscess was detected (data not shown). The final diagnosis was acute left tonsillitis with a left cervical abscess, with coexisting AR and a LE.

After transferring the patient to our hospital, his symptoms improved with antibacterial treatment alone and inflammatory markers normalized. Pharyngeal and blood cultures performed repeatedly in both hospitals were negative. A rapid antigen detection test for Group A *Streptococcus* performed in our hospital was also negative, and anti-streptolysin O was under the detection threshold. Based on these findings, the case did not fulfill the Modified Duke criteria (neither major nor minor criteria) for a definitive diagnosis of IE^5^ (S-Table 1, S-Table 2). Therefore, the antibiotic treatment was completed after 3 weeks.

By day 24, the patient had no recurrent fever or structural cardiac changes, and he was discharged. The risks of IE and cerebral infarction were explained to the patient and his family, and follow-up was performed without anti-thrombotic therapy. In the 2 years after discharge, no major changes in the size of the LE or related complications have been observed.

## Discussion

### Summary of pediatric cases

Reports of LEs in children are limited. To the best of our knowledge, this is the first pediatric case of LEs reported in Japan.

In 2018, Phillips et al. published the largest study to date on pediatric cases of LEs (n=12),^1^ while most other reports documented only one or a few cases.^3^ The patients in Phillips et al.’s study ranged from 5 months to 17 years old (median: 13.5 years old) and included 10 girls and 2 boys. Echocardiography was performed to investigate heart murmurs, syncope, abnormal electrocardiograms, and chest pain; however, AR was not detected in any of these 12 cases. Only 2 girls were initially diagnosed with LEs, while the other 10 cases were identified retrospectively through offline analysis of TTE data. One study reported an incidence of LEs of 2% among all pediatric patients who underwent TTE.^1^

In our case, a 9-year-old boy was diagnosed with LEs during an examination for the differential diagnosis of IE. AR was detected, although he remained asymptomatic. This finding suggests that LEs in children could be undiagnosed because they are frequently asymptomatic and might be overlooked in routine assessments.

### Comparison of pediatric cases of LEs with adults

LEs are more frequently observed in adults than in children, with an incidence of 5.5% in healthy subjects as shown by TEE. LEs are usually asymptomatic and, when detected, require regular follow-up. Among patients with cerebral infarction who undergo TEE, LEs have been found in 22% of cases, and they are considered a risk factor for cardiogenic cerebral infarction.^4,6–8^ In these patients, antithrombotic therapy, such as dual antiplatelet therapy or aspirin combined with warfarin, is recommended. However, in cases of repeated infarction, surgical resection is required.^2^

In children, the number of cases of LEs is limited. If patients have no symptoms, regular follow-up may be sufficient. In cases with a history of cerebral infarction, oral aspirin is recommended, and surgical resection may be considered in cases of recurrence.^1–3^

Although TEE is recognized as the gold standard for diagnosing LEs in adults, in pediatric patients, TTE is sufficiently informative and has the advantage of being noninvasive. Therefore, in children, TTE may be considered the preferred diagnostic method.^1^ However, TEE should be indicated in pediatric cases in which differentiation from IE is uncertain.

Currently, many adult cases of LEs are detected incidentally and remain asymptomatic. There is insufficient evidence regarding the necessity of immediate treatment for LEs, and no standardized therapeutic approach has been established. Therefore, the development of evidence-based treatment guidelines remains a critical future goal.^2,7^

LEs are rarely reported in children but may occur in those with left-sided valvular disease. Although not conclusive, preexisting AR may have been a predisposing factor for the development of the LE in this child, because LEs usually require a long time to form in otherwise healthy individuals.^9^ LEs are easily overlooked or can be misdiagnosed as vegetations associated with IE. Based on the fact that LEs can cause cerebral infarction, accurate detection through echocardiography is crucial, and affected patients with LE should undergo regular surveillance. Additionally, LEs should be considered a potential cause of stroke when evaluating children with stroke.

### Diagnosis

In pediatric cases of LEs, most are asymptomatic and incidentally detected and do not require surgical intervention. Therefore, the diagnosis is generally a clinical one based on the clinical course and echocardiographic findings, and histopathological confirmation for a definitive diagnosis is often unavailable.

When a vegetation on the aortic valve is incidentally detected in association with prolonged fever, as in the present case, physicians need to differentiate between IE, PFE, LE, and other conditions (differential diagnosis flow chart (S-Figure 1), S-Table 1, S-Table 2). In this case, all blood cultures obtained on three separate occasions at two institutions were negative. TTE and TEE were performed, and although IE is the most frequent entity and PFE is the next most common, the morphological features were not consistent with IE or PFE. A diagnosis of LE was made with the clinical course taken into account. However, this diagnosis was not confirmed histologically and was based primarily on echocardiographic findings and laboratory data, which represents a limitation of the diagnostic approach.

In summary, we report the first pediatric case of LEs in Japan. We have explained the risk of cerebral infarction from LEs to the patient and his family and continue to monitor him closely. To date, he has remained asymptomatic and is in a stable condition without medication, and the size of the LE has shown no progression on follow-up TTE. Our study highlights that, in children with valve structural abnormalities such as valvular regurgitation, the potential presence of LEs should be considered.

## Figures and Tables

**Table 1:** Laboratory findings

Peripheral blood test		Blood biochemical tests		venous blood gas analysis	
WBC	10800/μL	TP	7.8 g/dL	pH	7.372
Eo	0.5%	Alb	3.1 g/dL	pCO_2_	48.9 mmHg
Baso	2.6%	T.Bil	0.3 mg/dL	HCO_3_^–^	27.8 mEq/L
Neu	74.8%	D.Bil	0.1 mg/dL	BE	1.8 mEq/L
Lymp	14.8%	AST	20 U/L	Lactate	14.5 mg/dL
Mono	7.3%	ALT	14 U/L		
RBC	4.72×10^6^/μL	LD	176 U/L	Urinalysis	
Hb	13.3 g/dL	BUN	8.3 mg/dL	pH	7
Ht	39.4%	Cr	0.44 mg/dL	Urine protein	—
PLT	45.2×10^4^/μL	Na	135 mEq/L	Urine sugar	—
		K	4.5 mEq/L	Urinary occult blood	—
Coagulation fibrinolysis test		Cl	96 mEq/L	Urine ketones	—
PT	85%	CRP	3.65 mg/dL	Urine scum	
PT-INR	1.12	PCT	0.25 ng/mL	u-RBC	0
APTT	34 s	NT-proBNP	72 pg/mL	u-WBC	1~4
Fibronogen	602 mg/dL	IgG	2214 mg/dL		
FDP	5.1 μg/mL	IgM	163 mg/dL	bacterial culture	
D-dimer	1.3 μg/mL	CH50	52.2 U/mL	pharynx	(–)
		C3c	141 mg/dL	blood	(–)
		C4	17 mg/dL		
		ANA	<40 times		
		RF	<15 IU/mL		
		ASLO	≤10 IU/mL		

WBC, white blood cells; Eo, eosinophils; Baso, basophils; Neu, neutrophils; Lymp, lymphocytes; RBC, red blood cells; Hb, hemoglobin; Ht, hematocrit; PLT, platelets; PT, prothrombin time; INR, international normalized ratio; APTT, activated partial thromboplastin time; FDP, fibrin/fibrinogen degradation products; TP, total protein; Alb, albumin; T.Bil, total bilirubin; D.Bil, direct bilirubin; AST, aspartate aminotransferase; ALT, alanine aminotransferase; LD, lactate dehydrogenase; BUN, blood urea nitrogen; Cr, creatinine; Na, sodium; K, potassium; Cl, chloride; CRP, C-reactive protein; PCT, procalcitonin; NT-proBNP, N-terminal pro-B-type natriuretic peptide; Ig, immunoglobulin; CH50, total hemolytic complement activity; C3c, complement component 3c; C4, complement component 4; ANA, antinuclear antibody; RF, rheumatoid factor; ASLO, anti-streptolysin O; pCO_2,_ partial pressure of carbon dioxide; BE, base excess; u-RBC, urinary RBC.

## Meeting Presentation

The case was presented at the 60^th^ Annual Meeting of the Japanese Society of Pediatric Cardiology and Cardiac Surgery.
