# Supplementary material for: Lambl’s excrescences in a 9-year-old Japanese boy: differentiation from infective endocarditis
Source: Fujita Med J. 2026 May 14;12(3):255–9. doi: 10.20407/fmj.2025-049 (PMC13438285; doi:10.20407/fmj.2025-049)
Supplement: Supplementary file 1 [file fmj-12-255_s1.pdf]

S-Figure1

Differential Diagnosis Flowchart: PFE vs LE vs IE

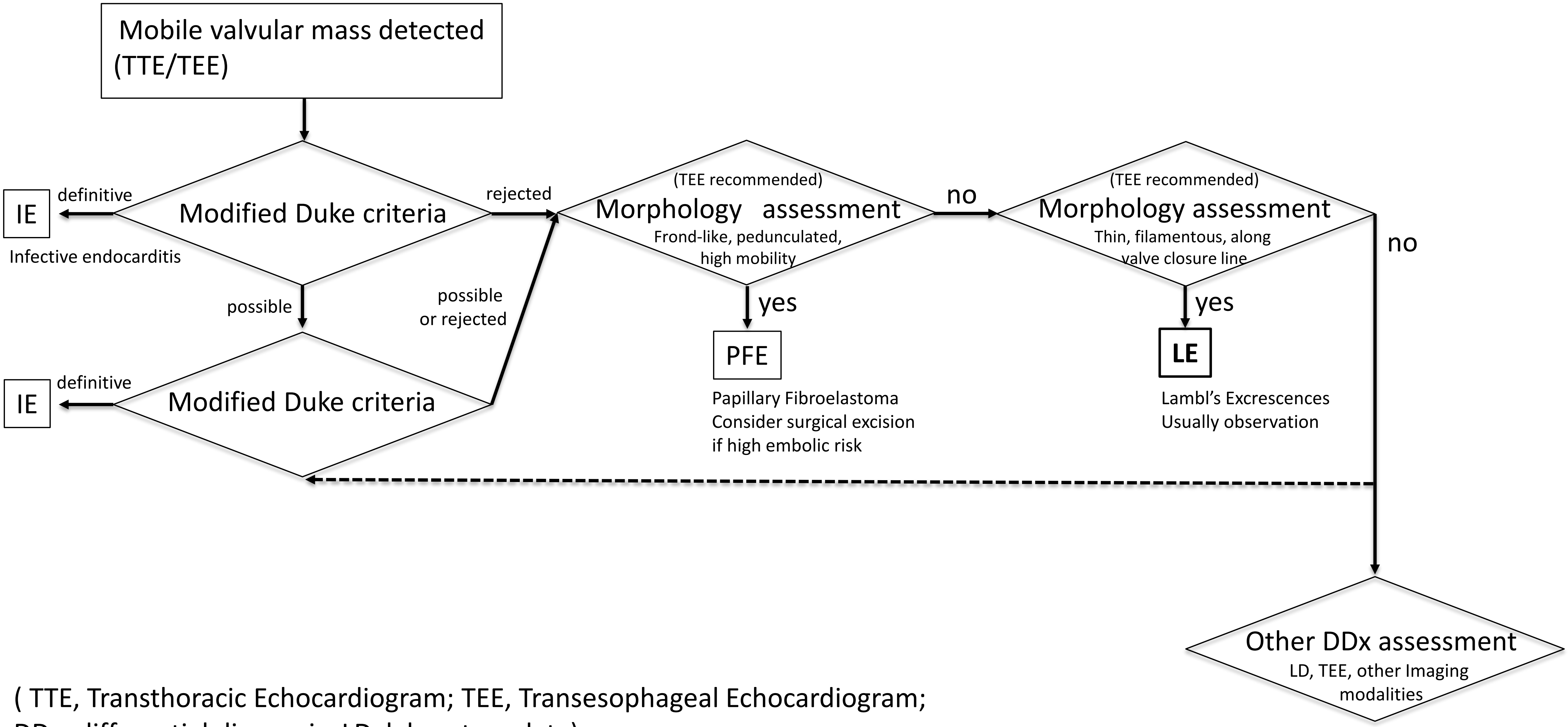

(TTE, Transthoracic Echocardiogram; TEE, Transesophageal Echocardiogram; DDx, differential diagnosis; LD, laboratory data)
