# Supplementary material for: Lambl’s excrescences in a 9-year-old Japanese boy: differentiation from infective endocarditis
Source: Fujita Med J. 2026 May 14;12(3):255–9. doi: 10.20407/fmj.2025-049 (PMC13438285; doi:10.20407/fmj.2025-049)
Supplement: Supplementary file 2 [file fmj-12-255_s2.pdf]

S-Table 1. Comparison of papillary fibroelastoma, Lambl's excrescences, and infective endocarditis 7), 8)

	**Lambl's excrescences (LE)** 1), 2)	**Papillary fibroelastoma (PFE)** 3), 4)	**Infective endocarditis (IE)** 5), 6)
Nature	Microscopic fibrous proliferation (degenerative)	Benign cardiac tumor	Infectious lesion (vegetation: platelets, fibrin, microorganisms)
Pathogenesis	Degenerative change	Neoplastic growth	Bacteremia → adherence to endocardium/valve
Frequency	Relatively common in adults	Second most common primary cardiac tumor	Occurs in children and adults
Typical location	Aortic valve, mitral valve	Aortic valve, mitral valve	Native/prosthetic valves, endocardium
**Gross morphology**	**Thin, filamentous strands**	**Frond-like, sea-anemone–like**	**Irregular, friable mass**
Size	Usually 1–4 mm	Usually 5–20 mm	Several mm to several cm
Stalk (pedicle)	Absent or indistinct	Clearly present	Often absent
Mobility	Mild to moderate	High	Often high
Echogenicity	Hyperechoic	Hyperechoic	Heterogeneous
Systemic symptoms	None	None	Fever, malaise, elevated inflammatory markers
Blood cultures	Negative	Negative	Often positive
Embolic risk	Low	Relatively high	High
Complications	Rare embolization	Stroke, myocardial infarction,	Heart failure, embolism,
Basic management	Observation	Consider surgical excision	Antibiotic therapy ± surgery
Prognosis	Good	Excellent after resection	Poor in severe cases

1) Saric M, et al. "Clinical and Echocardiographic Characteristics of Papillary Fibroelastomas: A 20-Year Experience and Comparison With Other Cardiac Tumors." J Am Coll Cardiol. 1999;34(5):1523–1530.
2) Tamin SS, et al. "Prognostic and Bioepidemiologic Insights From a Large Single-Center Cohort of Papillary Fibroelastomas." J Am Coll Cardiol. 2015;66(23):2424–2434.
3) Miller DV, et al. "Clinical, Echocardiographic and Pathologic Features of Lambl's Excrescences." Am Heart J. 1996;131(4):771–776.
4) Homma S, et al. "Lambl's Excrescences and Stroke." Stroke. 1999;30(2):449–455.
5) Li JS, et al. "Proposed Modifications to the Duke Criteria for the Diagnosis of Infective Endocarditis." Clin Infect Dis. 2000;30(4):633–638.
6) Baddour LM, et al. "Infective Endocarditis in Adults: Diagnosis, Antimicrobial Therapy, and Management of Complications." Circulation. 2015;132(15):1435–1486.
7) Maron BJ, et al. Braunwald's Heart Disease: A Textbook of Cardiovascular Medicine, 11th Ed.

S-Table 2. Modified Duke criteria for IE (with pediatric considerations)

Section	Category	Description
Major	Positive blood culture	Typical microorganisms from two cultures; persistent positivity; Coxiella burnetii or Phase I IgG ≥1:800
	Endocardial involvement	Vegetation, abscess, prosthetic valve dehiscence, OR new regurgitation
Minor	Predisposition	Predisposing heart disease or IV drug use
	Fever	Temperature ≥38°C
	Vascular phenomena	Emboli, septic pulmonary infarcts, mycotic aneurysm, Janeway lesions
	Immunologic phenomena	Glomerulonephritis, Osler nodes, Roth spots, RF
	Microbiological evidence	Positive culture not meeting major criterion
Diagnosis	Definite IE	2 Major OR 1 Major + 3 Minor OR 5 Minor
	Possible IE	1 Major + 1–2 Minor OR 3 Minor
	Rejected IE	Alternative diagnosis or no pathologic evidence
Pediatric	Predisposition	CHD, postoperative state, prosthetic material, intracardiac devices, CVC
	Echocardiography	Low threshold for TEE when TTE inconclusive

IgG, immunoglobulin G; IV, intravenous; RF, rheumatoid factor; IE, infective endocarditis; CHD, congenital heart disease; CVC, central venous catheter; TEE, transesophageal echocardiography; TTE, transthoracic echocardiography.
